# One Health, emerging infectious diseases and wildlife: two decades of progress?

**DOI:** 10.1098/rstb.2016.0167

**Published:** 2017-06-05

**Authors:** Andrew A. Cunningham, Peter Daszak, James L. N. Wood

**Affiliations:** 1 Institute of Zoology, Zoological Society of London, Regent's Park, London NW1 4RY, UK; 2 Ecohealth Alliance, 460 West 34th Street, New York, NY 10001, USA; 3 Department of Veterinary Medicine, Disease Dynamics Unit, University of Cambridge, Madingley Road, Cambridge CB3 0ES, UK

**Keywords:** One Health, ecosystem services, emerging infectious disease, zoonoses, wildlife disease, policy

## Abstract

Infectious diseases affect people, domestic animals and wildlife alike, with many pathogens being able to infect multiple species. Fifty years ago, following the wide-scale manufacture and use of antibiotics and vaccines, it seemed that the battle against infections was being won for the human population. Since then, however, and in addition to increasing antimicrobial resistance among bacterial pathogens, there has been an increase in the emergence of, mostly viral, zoonotic diseases from wildlife, sometimes causing fatal outbreaks of epidemic proportions. Concurrently, infectious disease has been identified as an increasing threat to wildlife conservation. A synthesis published in 2000 showed common anthropogenic drivers of disease threats to biodiversity and human health, including encroachment and destruction of wildlife habitat and the human-assisted spread of pathogens. Almost two decades later, the situation has not changed and, despite improved knowledge of the underlying causes, little has been done at the policy level to address these threats. For the sake of public health and wellbeing, human-kind needs to work better to conserve nature and preserve the ecosystem services, including disease regulation, that biodiversity provides while also understanding and mitigating activities which lead to disease emergence. We consider that holistic, One Health approaches to the management and mitigation of the risks of emerging infectious diseases have the greatest chance of success.

This article is part of the themed issue ‘One Health for a changing world: zoonoses, ecosystems and human well-being’.

## 1. Introduction

By the 1970s, the human burden of infectious diseases in the developed world was substantially diminished from historical levels, largely due to improved sanitation and the development of effective vaccines and antimicrobial drugs [1]. The emergence of a series of novel diseases in the 1970s and 1980s (e.g. toxic shock syndrome, Legionnaire's disease), culminating with the global spread of HIV/AIDS, however, led to infectious disease rising back up the health policy and political agendas [2]. Public concern about emerging infectious diseases (EIDs) has been heightened because of the perception that infectious diseases were previously under control, because of their often rapid spread (e.g. severe acute respiratory syndrome; SARS), because they often have high case fatality rates (e.g. Ebola virus disease) and because the development of drugs and vaccines to combat some of these (e.g. HIV/AIDS) has been slow and costly. By the 1990s, authors had begun to review similarities among these diseases and identify patterns in their origins and emergence [3,4]. Similarities included a skew to zoonotic pathogens originating in wildlife in tropical regions (e.g. Ebola virus), and that emergence was associated with environmental or human behavioural change and human interaction with wildlife (e.g. HIV/AIDS) or with domestic animals which had interactions with wildlife (e.g. Nipah virus) [5–7]. Emergence was found to be exacerbated by increasing volumes and rates of human travel and globalized trade [8].

By the end of the 1990s, the study of EIDs was a staple of most schools of public health, a key focus of national health agencies, a book topic and the title of a scientific journal [3]. Novel diseases continued to emerge, often from unexpected reservoirs and via new pathways. For example, between 1994 and 1998, three new zoonotic viruses (Hendra, Menangle and Nipah viruses) emerged from pteropodid bats in Australia and southeast Asia [9]. Each of these was transmitted via livestock (horses or pigs), and each belonged to the *Paramyxoviridae*. Around this time, emerging diseases were identified in a series of well-reported die-offs in wildlife, including canine distemper in African lions (*Panthera leo*) in the Serengeti, chytridiomycosis in amphibians globally, pilchard herpesvirus disease in Australasia and West Nile virus in corvids and other birds in New York [10–13]. Pathogens were also implicated for the first time in species extinctions, or near-extinctions, e.g. canine distemper in the black-footed ferret (*Mustela nigripes*), chytridiomycosis in the sharp-snouted day frog (*Taudactylus acutirostris*) and steinhausiosis in the Polynesian tree snail, *Partula turgida* [14–16]. Novel diseases and their emergence in people and wildlife were reviewed, and commonalities in the underlying causes of emergence discussed, in a paper published at the end of the decade [17]. Here, we re-examine some of the key conclusions of that paper, review how the field has progressed 17 years on and identify some of the remaining challenges to understanding and mitigating the impacts of disease emergence in and from wildlife.^1^

## 2. Disease threats to wildlife

Prior to 2000, wildlife diseases were mostly studied to improve zoo animal survival and welfare, with little published on the diseases of free-living wildlife unless they affected heavily hunted species (e.g. deer in North America) or were considered a threat to livestock health (e.g. tuberculosis, rinderpest). While non-infectious diseases had been widely recognized as important drivers of species declines (e.g. DDT poisoning of raptors [18,19]), only a small number of researchers investigated infectious disease as a factor in, often covert, wildlife population regulation [20,21]. The role of infectious diseases in mass mortality events or population declines was often considered controversial or secondary to other factors [22], and their role in species extinctions often disputed [23,24]. The first definitive identification of disease as a cause of species extinction was published in 1996 following the demise of the last population of the Polynesian tree snail *P. turgida* due to a microsporidian infection [16]. This added to evidence that infectious agents had caused the extinction in the wild of the black-footed ferret, the extinction of around one-third of Hawaiian honeycreepers and the slime mould-induced decline of eelgrass (*Zostera marina*) beds in the USA, leading to extinction of the eelgrass limpet (*Lottia alveus*) [14,25–27]. During the 1990s, wildlife mortality events caused by infectious diseases were reported in zoos, in wildlife translocation programmes and in other conservation programmes [28–32]. Perhaps the most important of these was the discovery of amphibian chytridiomycosis, caused by the chytrid fungal pathogen *Batrachochytrium dendrobatidis*, which was first recognized in the 1990s and has since been implicated in the decline or extinction of over 200 species of amphibian [11,15,33,34]. This disease continues to threaten amphibians globally and has been described as ‘the worst infectious disease ever recorded among vertebrates in terms of the number of species impacted, and its propensity to drive them to extinction’ [35].

Amphibian chytridiomycosis appears to have emerged contemporaneously in Australia and Central America, associated with large-scale die-offs and extinction events, although in retrospect it might have been causing amphibian mortalities and declines in North America prior to this [36]. Proving that a disease is a cause of population declines in wildlife requires longitudinal population and pathogen data, which are often very difficult to collect. Thus, a series of papers disputing the role of chytridiomycosis in amphibian declines ensued, with most suggesting that this disease either emerged secondarily to other factors, or that it was not the cause of declines/extinctions [37–40]. Long-term datasets have since been published which provide convincing evidence that amphibian chytridiomycosis alone can cause mass mortalities leading to population declines [41]. Policy measures to control amphibian chytridiomycosis, however, have been slow to be enacted, with the first international policy measure (listing of chytridiomycosis by the World Organisation for Animal Health) occurring in 2010 [42] and with the implementation of measures recognized to mitigate the spread of this disease still not being enacted by the international community [43].

Public and political reaction to the more-recent emergence of white nose syndrome (WNS) in North American bats provides evidence that the conservation implications of wildlife EIDs are becoming more widely accepted. The causative agent of WNS is the fungus *Pseudogymnoascus destructans* which colonizes the skin of a range of temperate-zone bats, often causing death during hibernation [44]. Only 1 year after the initial discovery of the disease in the USA in January 2007, visitors to bat caves across the country were being advised to reduce visits and to implement biosecurity measures, and by 2009, caves in over 20 states were closed to the public. The disease has been the focus of a series of grants, formation of multi-disciplinary research partnerships and significant efforts to identify pathogenesis, transmission pathways and potential control measures [45,46].

Although there is a growing recognition of the impact of pathogens on wildlife, the significance of infectious disease as a cause of historical extinctions is likely underestimated due to a previous relative lack of infectious disease focus and diagnostic capability [47]. Collaboration among ecologists, conservation biologists and veterinary pathologists is relatively recent and increased pathological and epidemiological involvement in studies of the causes of wildlife declines are critically needed to identify and understand disease threats to wildlife and how to mitigate them.

## 3. Zoonotic disease emergence from wildlife

In addition to identifying an apparently growing trend of disease threats to wildlife, Daszak *et al*. [17] highlighted wildlife as the source of a series of high-impact, recently emerging pathogens affecting people. These authors reiterated the widely proposed hypothesis that most emerging pathogens originate in wildlife and spillover into human hosts due to a range of ecological, demographic and socio-economic changes [1,3,48]. Prior to 2000, these wildlife-origin pathogens were known to include Ebola and Marburg virus, HIV-1 and HIV-2, Sin Nombre virus, Nipah, Hendra and Menangle virus, West Nile virus, *Borrelia burgdorferi* and others. Since then, other human diseases have emerged from wildlife, including Middle East respiratory syndrome (MERS) and different subtypes of avian influenza, and further advances have been made in our understanding of patterns of zoonotic disease emergence. A series of papers analysed a database of all known human EIDs and confirmed that the majority are of animal origin, with viruses being a particularly important group [49–52]. Further analysis of an updated version of this database identified that EIDs had increased in frequency (even accounting for increased numbers of researchers), with the proportion of those emerging from wildlife hosts increasing substantially over the last four decades of the twentieth century [53].

The emergence of bat-origin viral EIDs of people during the 1990s was highlighted by Daszak *et al*. [17]. Since then, it has been shown that bats are reservoir hosts of a striking number of zoonotic viruses, including high-profile pathogens with high case fatality rates, such as Nipah and Hendra paramyxoviruses, filoviruses, SARS-like coronaviruses and possibly also MERS coronavirus [54,55]. This led some authors to propose that bats harbour a disproportionate number of emerging zoonoses compared with other mammalian groups [55–57]: a hypothesis that has been supported by two separate analyses of mammal virus datasets [58,59]. Understanding why bats host so many zoonotic pathogens that cause lethal diseases in humans and how spillover from bats to humans occurs is important in order to control these, and possibly as-yet-undiscovered, diseases [58,60–63].

## 4. Drivers of disease emergence

There are likely to be multiple causes of novel disease emergence, but the human-mediated transport of pathogens (often in infected hosts) or vectors across geographical or ecological boundaries, a process termed ‘pathogen pollution’, has been identified as a major driver of this in wildlife [64] and also in plants [65]. The anthropogenic spread of pathogens has been responsible for the emergence of a series of high-profile wildlife EIDs, including the two known agents of amphibian chytridiomycosis, *B. dendrobatidis* and *B. salamandrivorans* [66,67]. Subsequent research indicates that this is only part of the story, as it appears that the global pandemic lineage of *B. dendrobatidis* arose from a single hybrid origin via an ancestral meiosis, possibly via the anthropogenic mixing of allopatric lineages [68,69]. There is a substantial volume of research that shows how, once evolved, this virulent lineage has been introduced globally via the international trade in amphibians and via the human-assisted introduction of invasive species [66,70–75].

In recent years, a body of literature has developed the concept of the ecosystem service of disease regulation. While still controversial, and probably not universal [76], this proposes that natural biodiversity limits the exposure and impact of many pathogens, including those that are zoonotic, through a dilution or buffering effect, thus limiting opportunities for pathogen spillover from wildlife to people [77]. When biodiversity is depleted (usually by human activities), this ecosystem service is impaired and zoonotic pathogens are more likely to emerge, as has been shown for hantavirus [78] and for *B. burgdorferi*, the causative agent of Lyme disease [79,80]. Also, alteration of species complements (again, usually due to anthropogenic impacts), rather than loss of biodiversity *per se*, can alter infection dynamics and lead to increased zoonotic disease risk [81]. Our understanding of the interactions between ecosystem change, disease regulation and human well-being, however, is in its infancy.

Almost 20 years since the threats to conservation and human health that wildlife EIDs represent was first highlighted, there has been little effort to put in place policies to reduce risk. Detecting and preventing the importation of infected hosts is widely used to prevent importation of many domestic animal diseases of economic or public health importance. Some countries even enact this principle for the movement of people, whereby they conduct (often cursory) surveillance for infected persons arriving at their international borders, particularly during human pandemics [82,83]. The World Health Organisation provides guidance and training on this through its International Health Regulations (http://www.who.int/ihr/en/). Rules and regulations for international trade, including of animals and their products, are created and enforced by the World Trade Organisation (WTO), which has the remit of ensuring ‘that trade flows as smoothly, predictably and freely as possible’ (www.wto.org). The WTO agreement on sanitary and phytosanitary measures was enacted on 1 January 1995 with the aim of protecting human, animal and plant life from disease-causing agents. While countries have discretion in what should be included, they are guided by the World Organisation for Animal Health (OIE) list of diseases of international importance. Although the OIE has a remit of protecting biodiversity, only two pathogens are listed for this purpose: *B. dendrobatidis* and *Ranavirus* [42]. Most countries, therefore, use import controls to only protect against domestic animal diseases of obvious public health or economic importance, such as rabies and foot and mouth disease; diseases restricted to wildlife are not included even when OIE-listed.

In addition, trade agreements often prohibit barriers to international animal movements for the purposes of infectious disease control. For example, countries within the European Union have little ability to prevent the spread of pathogens via within-EU trade unless as part of a specific EU disease control programme. Even where technically legal under WTO rules, there appears to be reluctance by countries to unilaterally impose restrictions on non-listed diseases in case they create an economic disadvantage or are subsequently found to be in breach of international trade regulations. It is possible that the international spread of amphibian chytridiomycosis would have been reduced if such measures had been implemented for this disease [43]. Perhaps learning from this, in January 2016, the USA banned the importation of salamanders following the emergence of *B. salamandrivorans* in order to protect native wildlife from this novel pathogen [84]. Such protective action was enacted relatively rapidly following the discovery of *B. salamandrivorans* as a novel lethal fungus infecting and killing captive and wild salamanders in Europe [67,85,86]. Hopefully, this will open the doors to the imposition of trade controls for other diseases and by other nations in order to protect biodiversity from the anthropogenic spread of pathogens.

Challenges remain to understanding the wildlife origins of zoonotic EIDs. It is often difficult, time-consuming, logistically challenging and very expensive to identify the origins of newly emerged pathogens of humans. For example, viruses similar to HIV/AIDs were discovered in non-human primates in the early 1980s, but identification of the true progenitor viruses in chimpanzees took almost a decade of additional research [87]. Similarly, the origins of Ebola and Marburg viruses have been investigated for over 30 years. To date, however, despite indications that bats are the natural reservoir hosts of these viruses, clear evidence has only been found for Marburg virus infection in bats in limited locations [88–90]. Identifying putative reservoir host(s) is just the beginning. In order to identify actions to prevent or mitigate future zoonotic spillover, both an understanding of the ecology of the pathogen in its natural host(s) and of human–host interactions are required [63]. For example, substantial efforts have been conducted to understand immunological, behavioural and ecological characteristics of bats as part of a strategy to control zoonotic spillover from bats [91–93]. Long-term, multi-disciplinary studies that systematically investigate the ecology of zoonotic pathogens in their wildlife hosts along with the risk characteristics for spillover are critical to better predict and prevent future pandemics [63]. Such a study, which included years of field data collection on fruit tree distribution, pig farm management, viral dynamics and satellite telemetry of fruit bats, analysis of climate trends, experimental infection of bats under BioSafety Level-4 conditions and mathematical modelling of virus infection dynamics, identified the intensification of the pig industry as the driver of the zoonotic emergence of Nipah virus in Malaysia [94]. These results informed government policies to separate pigs from bats via the removal of fruit trees from pig farms and the relocation of farms away from forested areas [95], since when no further Nipah virus disease outbreaks have occurred in Malaysia.

## 5. Endemic zoonoses from wildlife

EID events have been the focus of intense research over the past two decades, even though the numbers of people diagnosed with them are often relatively small. This disproportionate focus on EIDs probably relates to the dislike of human society for uncertainty, or put more simply, fear of the unknown. This may lead to perverse scenarios in which fear of disease can have a greater impact than the direct impact of the outbreak itself. For example, during a recent Ebola virus epidemic in West Africa, more people are estimated to have died from malaria due to their avoidance of healthcare facilities, where they feared they might catch Ebola, than the thousands that died from the virus itself [96].

Indeed, when one considers the overall impact of zoonotic diseases on the human population, the largest (diagnosed) burden is associated with well known and fully recognized (in the industrial north), but neglected, diseases such as brucellosis, rickettsioses and Rift Valley fever [97]. This predictable burden falls heavily on the global poor—poverty being the major risk factor for most zoonoses, which in turn causes some communities to suffer disproportionately from the burden of zoonotic disease [97]. The neglect of such diseases includes diagnostic neglect (and confusion with other conditions such as malaria [98]) and historic and current research neglect; all of which feeds into therapeutic neglect. The delivery of the United Nations sustainable development goals, which should result in much reduced poverty and improved health, will in themselves reduce the substantial burden of zoonotic disease.

## 6. Whither One Health

One Health is the term used when approaches to tackling disease (particularly zoonoses) consider all components that might lead to, or increase, the threat of disease. These include environmental and ecological/wildlife components as well as domestic animal and human factors. The last encompasses behavioural as well as medical issues, including cultural, political and other socio-economic drivers that might result in disease occurrence or spread. The review by Daszak *et al*. [17] was perhaps the first ‘One Health’ review of emerging diseases, in that it brought together veterinary, ecological, conservation and human medical perspectives on disease emergence. The field of One Health has expanded substantially since 2000, diversifying to produce new journals, such as *One Health*, *EcoHealth* and *The Lancet Planetary Health*, the One Health Platform, the International Association of Ecology and Health, the Planetary Health Alliance and a series of One Health institutions in the USA, Europe, Australia and increasingly also in developing countries. The success of this multi-disciplinary approach has been driven largely by the synergistic impact of combining detailed and logistically challenging field sciences (e.g. ecology, field biology) with analytical approaches (e.g. epidemiological modelling, pathogen phylogenetic analysis) and laboratory science (e.g. serology, pathogen diagnostics, immunology). Challenges remain, however. Importantly, while the conservation, ecological and veterinary professions are increasingly engaged with One Health, substantial elements of the medical profession are not aware of, or involved in, this approach.

Despite their neglect, a number of zoonotic diseases are eminently controllable or manageable by One Health approaches, including infectious causes of abortion in livestock, which frequently result in febrile human disease, and human rabies transmitted via dog bites. Control or prevention is best achieved through integrated public health, veterinary medicine, animal management and ecological approaches. One particular challenge for this is in the case of some zoonotic infections that do not cause clinical signs in their animal hosts, one of the most common examples of which is *Campylobacter* spp. infection of poultry, which globally is the most frequent cause of food poisoning in humans [99]. Is it, then, the responsibility of farmers and vets to ensure that people do not become infected, or of public health practitioners or the general public through improved kitchen hygiene and behaviours? Here, this would involve reduced infection of poultry (the role of farmers and veterinarians), reduced contamination of meat (the responsibility of veterinary public health workers) and preventive measures in the kitchen (hygiene and proper cooking), which are the domain of public health workers and the public [99].

One Health approaches are required at the policy and governance levels, too. Responsibility for preventing and treating zoonotic disease, in both a developing and developed world setting, for example, often falls in between government Ministries of Health and Agriculture (and for wildlife, Ministries of Environment and Forestry) and this can structurally prevent the simplest of solutions from being implemented. An important example is rabies in humans transmitted through dog bites which kills around 60 000 people annually [100] and causes fear in many more in rabies endemic regions. The disease is easily preventable (and arguably open to eradication) through repeated annual or biannual mass vaccination of dogs [101]. In many countries with a high burden of rabies in dogs, considerable sums are spent by the public and Ministries of Health annually on post-exposure prophylaxis (PEP—often given after dog bites whether or not the animal was known to be rabid). The expense of this repeated treatment usually dictates that far more is spent on treatment than would be required to vaccinate all dogs in the same region. However, in many countries, the dog is regarded as a pest and not an agricultural animal for which Ministries of Agriculture have responsibility. In others, the dog does fall under the Agricultural Ministry, but these Ministries are typically far less well resourced compared with Ministries of Health, thus rabies, which does not relate to food animals, is not prioritized. The obvious solution is for a synergized One Health approach with the Ministries of Health supporting prophylactic vaccination programmes for dogs delivered by their typically far less well-resourced Ministries of Agriculture. This, however, rarely seems to happen and continued expenditure on bite management and PEP continues. One Health programmes addressing rabies have been extremely successful when appropriately resourced [102,103]; however, they often fail to influence national government policy and are rarely adopted long term [104].

## 7. Policies for prevention and control

In addition to the high costs of dealing with endemic zoonoses, such as rabies, emerging and re-emerging zoonoses can have substantial economic impacts. The cost implications of zoonotic EIDs were highlighted by Daszak *et al*. [17] as a rationale for policy measures, but methods for calculating the economic consequences of disease emergence have not advanced in the interim. Despite clearly high financial impacts associated with some EIDs, few detailed economic analyses of their impact have been undertaken. Estimates of the cost of the 2003 SARS outbreak, for example, range from $5 to $50 billion, while the true costs of most EIDs have never been estimated [105]. Pike *et al.* [105] approached the problem of disease emergence in the same way as the climate change phenomenon. They used the increasing frequency of emerging disease events reported by Jones *et al*. [53] to analyse two strategies to deal with the rising costs of EIDs over time: adaptation, whereby we adopt a business-as-usual approach and continue to cause increased EID events, then target control programmes after emergence; and mitigation, whereby we deal with the underlying drivers (e.g. wildlife trade, deforestation) and reduce the frequency of EID events. Pike *et al.* [105] show that mitigation strategies are more cost effective in the long term, with a 10-fold return on investment, and that these need to be enacted on a global scale within the current generation or the cost of EIDs becomes unaffordable.

What would these global strategies entail? We highlight three approaches. First, a series of emerging diseases have been linked to the wildlife trade, or consumption of wildlife (e.g. SARS, Ebola). The health implications of the trade in wildlife have not been widely used to implement controls, or advocate for reduction in consumption, and may be a more effective message than its conservation impacts. This needs to be done judiciously, however, as disease spillover is a rare event and both bushmeat hunters and consumers will be wary of public health messages that do not fit with their experiences [93,106].

Second, a revision of an earlier analysis of global drivers of disease emergence [53] shows that land-use change correlates strongly with the emergence of zoonoses from wildlife (P Daszak 2017, unpublished observation). In Malaysia, analyses of the economic cost of diseases that emerge due to land conversion for palm oil production (e.g. malaria, leptospirosis) are currently being used to advise industry where to reduce long-term impact. Identifying land-use changes that lead to disease emergence informs policies for mitigation strategies. This could be done, for example, via the incorporation of wildlife and zoonotic disease threats in environmental impact studies, an approach for the prevention of disease emergence suggested by Daszak *et al*. [17].

Third, targeted global surveillance programmes to identify novel pathogens of zoonotic potential before they emerge may increase our capacity to reduce their risk of emergence. For example, a series of laboratories now specialize in identifying novel viruses from wildlife hosts, e.g. bats [107–113]. The USAID Emerging Pandemic Threats programme specifically targets emerging disease hotspots to identify novel viruses from bats, rodents and primates, to characterize high-risk behaviours in people and to identify potential mitigation strategies [60]. While these programmes have already identified over 1000 new viruses from viral families with known zoonoses in the last few years, challenges remain in how to identify those with the highest (or any) risk of zoonotic emergence. This indicates that a change in approach is required, building on rapidly expanding databases of pathogen sequences, phenotypic characteristics and host–pathogen interactions. For example, the rapid incorporation of novel viral sequences into diagnostic tests may lead to more rapid identification of related, previously unknown, pathogens that emerge in outbreaks. Using this approach, combined with a One Health perspective that targets the underlying drivers of emergence, could result in the identification of pathogens that already are spilling over from wildlife hosts sporadically at low levels, enabling measures to be taken to reduce pandemic risk.

## 8. Conclusion

Since the synthesis paper by Daszak *et al*. [17] highlighted emerging disease threats of, and from, wildlife and the main drivers underlying these, further advances have been made in our understanding of the origin, size and potential scope of these threats. Endemic zoonoses, however, continue to be relatively neglected, often with a lack of local and international realization of the extent to which they impact human health and well-being. This is partly due to issues surrounding local capacity and knowledge and partly because, unlike EIDs, they are not seen as a threat to people in the developed world. Both EIDs and endemic zoonoses, however, can be tackled using a One Health approach, including the identification and mitigation of human activities that lead to disease emergence and spread. One Health approaches to dealing with disease threats from and to wildlife are still relatively young and untried, but all evidence points to them being most successful and cost-effective if developed and implemented in full by all relevant parties, including policy-makers and the medical profession.

## Endnote

1 In this paper, when we discuss wildlife, we refer to non-domesticated animals regardless of taxon.
